# Some questions about preadmission metformin use and mortality in patients with sepsis and diabetes mellitus

**DOI:** 10.1186/s13054-019-2392-y

**Published:** 2019-03-25

**Authors:** Jiarong Ye, Qianrong Liang, Xiaotu Xi

**Affiliations:** 1 The Second Affiliated Hospital of Guangzhou University of Chinese Medicine and Guangdong Provincial Hospital of Chinese Medicine, No. 111, Dade Road, Yuexiu District, Guangzhou, 510120 China; 2 Guangdong University of Foreign Studies, No. 2, Baiyun Avenue North, Baiyun District, Guangzhou, 510420 China

We read with great interest the article by Liang et al. recently published in *Critical Care* [1]. The authors concluded that there is an association between metformin use prior to admission and lower mortality in septic adult patients with diabetes mellitus. Although it sounds scientific, there are some questions worthy of our attention.

First, the article overlooks the association of mortality and dysglycemia itself in septic patients. So far, the impact of serum glucose levels on disease severity and outcome in patients with sepsis remains controversial. One study [2] pointed out that there were no significant differences in disseminated intravascular coagulation, Sequential Organ Failure Assessment or Acute Physiology and Chronic Health Evaluation II scores, and mortality rates between septic patients with or without pre-existing diabetes, whereas the studies by Tayek and Tayek [3] and Chao et al. [4] indicated that while diabetes mellitus seems to be a protective factor in sepsis patients, hyper- or hypoglycemia status on admission, and increased blood glucose variation during hospital stays, was independently associated with increased odds ratio of mortality. Thus, it did not seem persuasive that metformin use could completely predict outcomes without adjusting for the covariate of diabetes. This requires high-quality research to further demonstrate the relationship between diabetes and mortality in patients with sepsis.

Second, the article pointed out that the heterogeneity may be derived from many causes, such as the small sample sizes, the different initial lactate levels, and the use of other antidiabetic medications. However, differences in the Simplified Acute Physiology Score II, Acute Physiology and Chronic Health Evaluation II (APACHE) II score, and Sequential Organ Failure Assessment (SOFA) score baselines may also lead to statistical heterogeneity in the five studies included [5]. Thus, it is necessary to adjust Simplified Acute Physiology Score II, APACHE II score, and SOFA score for statistical analysis in this article as these details could change the final results.

Third, the authors may have made a mistake. A total of five cohort studies were included in the meta-analysis, but in the discussion section, the authors mentioned that only four studies met their inclusion criteria.

The last, we appreciate Liang et al. for this meaningful research which provides us with the relationship of preadmission metformin use and mortality in patients with sepsis and diabetes mellitus, though some minor details need to be discussed and amended. In addition, more high-quality original studies should be conducted to confirm and validate this association.
